# Deep learning-based multimodal approach for non-invasive prediction and prognostic analysis of immune and angiogenic biomarkers in extrahepatic cholangiocarcinoma

**DOI:** 10.3389/fimmu.2025.1658122

**Published:** 2026-01-13

**Authors:** Jiong Liu, Zhitao Cheng, Ruidan Yang, Yinfei Fan, Yue Shu, Yong Tang, Jian Shu

**Affiliations:** 1 Department of Radiology, The Affiliated Hospital of Southwest Medical University, Luzhou, Sichuan, China; 2 Precision Imaging and Intelligent Analysis Key Laboratory of Luzhou, Luzhou, Sichuan, China; 3 School of Computer Science and Engineering, University of Electronic Science and Technology of China, Chengdu, China; 4 Department of Oncology, The Affiliated Hospital of Southwest Medical University, Luzhou, China; 5 Institute of Intelligent Chinese Medicine, Chongqing University of Chinese Medicine, Chongqing, China

**Keywords:** deep learning, extrahepatic cholangiocarcinoma, magnetic resonance imaging, programmed death ligand 1, vascular endothelial growth factor

## Abstract

**Objective:**

This study aims to develop a deep learning (DL)-based multimodal framework that integrates magnetic resonance imaging (MRI), clinical, and laboratory data to predict programmed death ligand 1 (PD-L1) and vascular endothelial growth factor (VEGF) expression in Extrahepatic cholangiocarcinoma (eCCA) patients and assess the prognostic value.

**Methods:**

A retrospective cohort study involving 96 patients with eCCA was conducted across two institutions. A total of 16050 raw MRI images (11505 T1WI, 2371 T2WI, 2372 DWI) and 1570 tumor-containing images (990 T1WI, 289 T2WI, 291 DWI) were analyzed. Radiomic feature extraction was performed manually segmented tumor regions from MRI scans. The multimodal DL framework integrated DL features extracted from images and radiomic features as well as clinical-laboratory features through a repeated attention mechanism. Prognostic stratification was performed using Cox regression analysis to predict overall survival (OS) and evaluate the clinical utility of the model.

**Results:**

The DL framework demonstrated moderate predictive performance for PD-L1 expression (AUC = 0.71) and good predictive capability for VEGF expression (AUC = 0.85) in the test cohort. The combination of DL-based imaging features and radiomic data outperformed single-modality approaches. Prognostic analysis revealed significant associations of model-predicted PD-L1 and VEGF expression with OS in eCCA patients. The Cox model-based nomogram demonstrated significant survival stratification (p = 0.006), with performance comparable to traditional immunohistochemistry-based methods.

**Conclusion:**

Our findings highlighted the potential of integrating DL and radiomics for non-invasive, preoperative biomarker profiling, offering a promising tool for personalized treatment strategies and improved clinical decision-making in eCCA.

## 1 Introduction

Extrahepatic cholangiocarcinoma (eCCA) is a rare malignant epithelial tumor originating from the perihilar and distal bile ducts. It is characterized by high invasiveness, heterogeneity, and poor prognosis (1). Accounting for 70%–90% of all CCA cases, eCCA has a higher global incidence compared to intrahepatic cholangiocarcinoma (iCCA), at approximately 1.02 per 100,000 population (2, 3). Due to subtle and non-specific early symptoms, approximately 80% of eCCA patients are diagnosed at advanced stages, thus missing the optimal window for curative surgery (4). Even for those undergoing R0 resection, the recurrence rate remains as high as 75%, and the five-year overall survival (OS) rate is only about 5% for patients with advanced disease (5, 6). Given the high mortality rates of eCCA, there is an urgent clinical need to develop novel therapeutic approaches and identify effective biomarkers capable of predicting patient prognosis and treatment response.

In recent years, immunotherapy and targeted therapy have emerged as promising options for advanced eCCA. Programmed death ligand 1 (PD-L1), a critical molecule in tumor immune evasion, inhibits T-cell activation by binding to programmed death protein 1 (PD-1), allowing tumor cells to evade immune surveillance (7). Similarly, angiogenesis, mediated by vascular endothelial growth factor (VEGF), plays a crucial role in tumor growth and metastasis. Elevated VEGF expression has been found strongly associated with poor prognosis in eCCA, making it a potential biomarker for anti-angiogenic therapies (8–10). Furthermore, the interaction between angiogenesis and tumor immune microenvironment (TIME) suggests that a dual blockade of PD-L1/PD-1 immune checkpoints and VEGF-mediated angiogenesis may synergistically improve treatment outcomes (11–13).

Despite these advancements, the preoperative identification of PD-L1 and VEGF expression in eCCA remains challenging. Immunohistochemical staining, currently the gold standard for biomarker detection, relies on tumor specimens obtained through invasive procedures, which limits its practicality for repeated or dynamic assessments. Magnetic resonance imaging (MRI), a widely used modality for preoperative diagnosis and staging of eCCA (14), traditionally focused on morphological features, limiting its utility in capturing molecular or immune characteristics. However, advances in radiomics and deep learning (DL) have demonstrated great potential for the non-invasive prediction of molecular biomarkers in various tumor types (15, 16). By extracting and analyzing high-dimensional data from imaging modalities, DL models can uncover subtle patterns related to the tumor microenvironment, facilitating comprehensive biomarker accessment without invasive procedures.

This study aims to develop a DL-based multimodal framework that combines MRI radiomic features with clinical and laboratory data to predict PD-L1 and VEGF expression as well as survival prognosis in eCCA patients. By providing a non-invasive, preoperative biomarker assessment tool, this study aims to improve personalized treatment strategies and predict patient survival outcomes, ultimately enhancing clinical decision-making and patient prognosis.

## 2 Materials and methods

### 2.1 Patient enrollment

**Figure 1:**
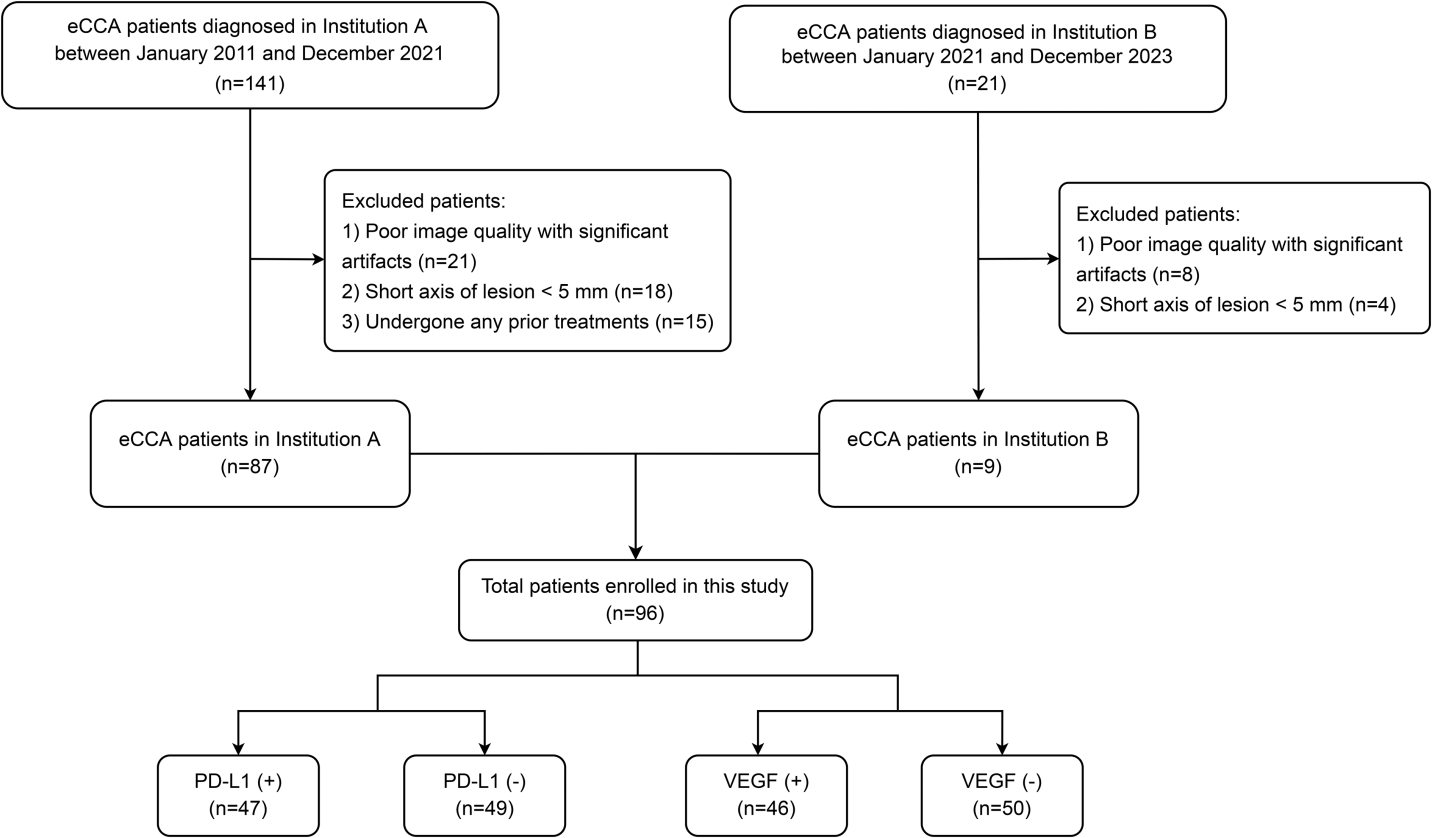
Flowchart of the patient selection process.

A retrospective analysis was conducted in eCCA patients diagnosed at Institution A between January 2011 and December 2021, and at Institution B between January 2021 and December 2023. The inclusion criteria were: (1) postoperatively confirmed eCCA through pathological tissue biopsy; (2) availability of complete preoperative multi-sequence MRI images; and (3) comprehensive clinical data. The exclusion criteria were: (1) poor image quality with significant artifacts; (2) lesions too small (short axis < 5 mm) to accurately delineate tumor boundaries; and (3) patients who had undergone any prior treatments (such as surgery, chemotherapy, or interventional therapy) before MRI scanning. A total of 96 eCCA patients meeting the inclusion and exclusion criteria from both centers were included (**Figure 1**). Due to the relatively small sample size from each center, patient data from both centers were combined into a unified dataset to increase the reliability of statistical inferences. This study was approved by the Ethics Committee of the Institution A (KY2023041), and the requirement for informed consent was waived.

In this study, 15 common clinical features and laboratory indicators were analyzed, including patient age, gender, tumor location, and key biomarkers such as carbohydrate antigen 19-9 (CA19-9), CA50, carcinoembryonic antigen (CEA), alpha-fetoprotein (AFP), and ferritin (FER). Additionally, liver function markers including alkaline phosphatase (ALP), γ-glutamyl transpeptidase (γ-GTP), aspartate aminotransferase (AST), alanine aminotransferase (ALT), total bilirubin (TBIL), and direct bilirubin (DBIL) were included, along with systemic inflammatory markers such as the neutrophil-to-lymphocyte ratio (NLR).

### 2.2 Evaluation of PD-L1 and VEGF expression

**Figure 2:**
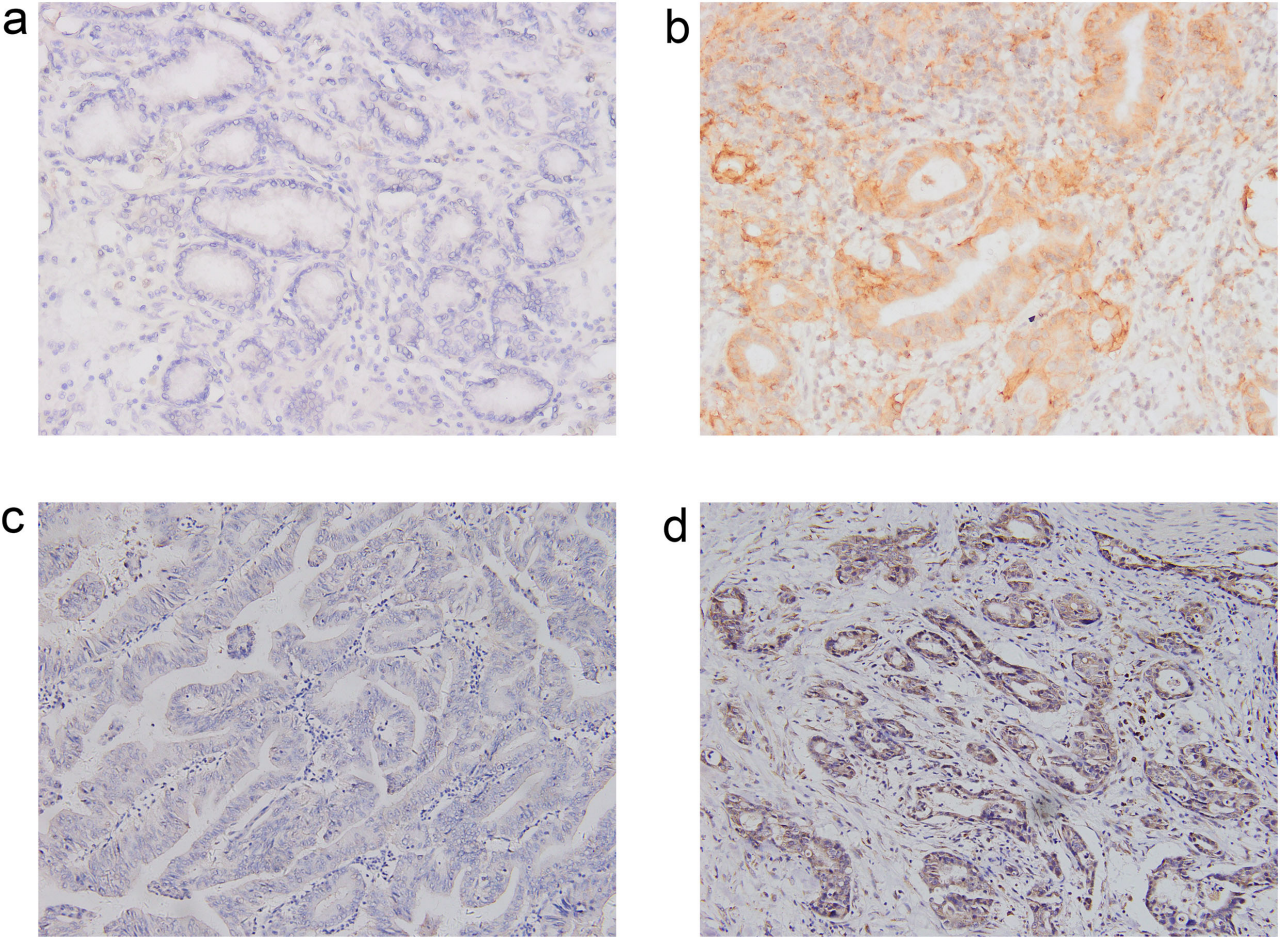
Immunohistochemical Staining (×200 Magnification). **(a)** PD-L1 Negative; **(b)** PD-L1 Positive; **(c)** VEGF Negative; **(d)** VEGF Positive.

The expression of PD-L1 and VEGF was detected using the immunohistochemistry (IHC) Envision method (**Figure 2**). The primary antibodies used were the PD-L1 SP142 antibody (Beijing Zhongshan Golden Bridge Biotechnology Co., Ltd.) and the VEGF antibody (Luzhou Keyang Biotechnology Co., Ltd.). The evaluation of both markers was interpreted by pathologists with over 10 years of experience in pathological diagnosis.

PD-L1 expression was quantified using the Combined Positive Score (CPS), which was calculated by dividing the total number of PD-L1-positive tumor cells and surrounding immune cells by 100 tumor cells. Given the low expression rate of PD-L1 in eCCA patients and in reference to related studies on other malignancies, a CPS ≥ 1 was classified as weakly positive, while a CPS ≥ 50 was categorized as strongly positive. Both groups were collectively defined as PD-L1 positive, whereas patients with a CPS < 1 were classified as PD-L1 negative.

VEGF expression was determined by cytoplasmic staining ranging from light yellow to brown, and the percentage of positive cells relative to the total number of cells was calculated. No positive cells (0%) indicated no VEGF expression. A positive cell percentage below 25% was considered weakly positive, between 25% and 50% was moderately positive, and more than 50% was strongly positive. Finally, patients with no or weak VEGF expression were classified as VEGF negative, while others were categorized as VEGF positive.

### 2.3 MRI protocol

Institution A conducted standard upper abdominal imaging using a 3.0T whole-body superconducting MRI system (Achieva, Philips, Netherlands) equipped with a 16-channel phased-array torso coil. Similarly, Institution B performed comparable scans utilizing a 3.0T whole-body superconducting MRI system (Skyra, Siemens, Germany) with an 18-channel phased-array torso coil. The imaging protocols in both institutions were designed to comprehensively cover the biliary system. Core sequences included axial T1-weighted imaging (T1WI), axial T2-weighted imaging with spectral attenuated inversion recovery (T2WI-SPAIR), and axial diffusion-weighted imaging (DWI), among others. Detailed scanning parameters are summarized in **Table 1**.

**Table 1:** Scanning parameters for upper abdominal MRI in both institutions.

Parameters	Institution A			Institution B		
	T1WI	T2WI	DWI	T1WI	T2WI	DWI
Repetition time (ms)	3.1	1610	934	3.32	1100	5600
Echo time (ms)	1.44	70	52	1.3	95	56
Slice thickness (mm)	3	7	7	3.0	5	6
Slice spacing (mm)	1.5	1	1	0.6	1	1.2
Field of view (mm^2^)	280×305	280×305	280×305	308×380	308×380	308×380
Matrix size	244×186	176×201	100×124	240×320	256×320	134×134
Flip angle (°)	10	90	90	9.0	145	–
B-value (s/mm^2^)	–	–	0 / 800	–	–	50 / 400 / 800

### 2.4 Lesion segmentation

**Figure 3:**
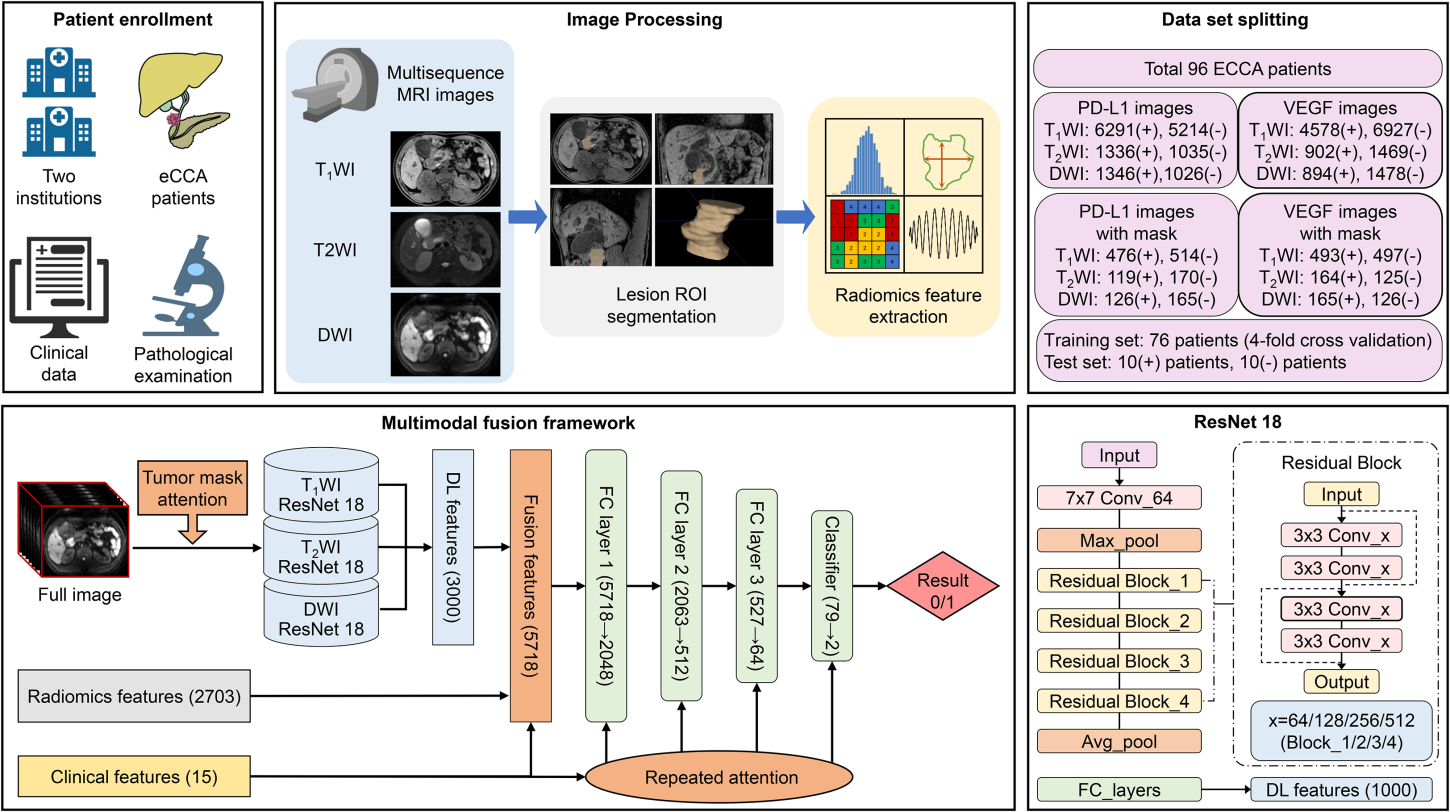
Study schematic.

All MRI images were retrieved and stored in the Picture Archiving and Communication Systems (PACS), and subsequently imported into the open-source software 3D-Slicer (version 4.11, https://www.slicer.org/). Two radiologists (R1 and R2), both with more than five years of experience, confirmed the lesions and established the criteria for delineating the volume of interest (VOI) for the whole tumor: (1) the tumor was outlined slice-by-slice along its borders in the three imaging sequences; (2) internal tumor components such as vessels, necrotic cysts, and hemorrhage were included; (3) surrounding vessels and adjacent dilated bile ducts were excluded (**Figure 3**).

### 2.5 Radiomic feature extraction and consistency assessment

Before feature extraction, all images were resampled into 3mm×3mm×3mm voxel size to standardize the imaging data. Additionally, the “bin width” parameter was set to 25, and the gray-level co-occurrence matrix (GLCM) feature was forced to symmetry. Using the Pyradiomics package, radiomic features were extracted from the three MRI sequences for each patient, including 19 first-order statistical features, 16 three-dimensional shape features, 75 texture features, and high-order features derived from wavelet and LoG filters. All features adhered to the Image Biomarker Standardization Initiative (IBSI) guidelines. In total, 851 T1WI features, 1316 T2WI features, and 1316 DWI features were extracted for each patient (**Figure 3**).

To assess the stability of the extracted radiomic features, images from 20 randomly selected eCCA patients were used for consistency testing across the three MRI sequences. Two radiologists (R1 and R2) outlined the tumors independently, and radiologist R1 repeated the delineation after one week. The intraclass correlation coefficient (ICC) was calculated using Python (version 3.7, https://www.python.org/), with a two-way random-effects model and absolute agreement to evaluate both inter-observer and intra-observer reliability. Radiomic features with ICC values ≥ 0.75 for both inter-observer and intra-observer assessments were retained. This stability assessment was performed independently for each of the three MRI sequences (T1WI, T2WI, DWI). After ICC analysis, 502 T1WI features, 1113 T2WI features, and 1088 DWI features were retained, resulting in a total of 2703 radiomic features.

### 2.6 Multimodal DL framework construction

Our DL architecture integrated three distinct data modalities: multi-sequence MRI images, radiomic features, and clinical parameters. For imaging analysis, we employed the ResNet18 backbone to extract high-dimensional features from both the original images and their corresponding segmented tumor regions across all sequences. A total of 16050 raw MRI images (11505 T1WI, 2371 T2WI, 2372 DWI) and 1570 tumor-containing images (990 T1WI, 289 T2WI, 291 DWI) were analyzed. Patient-specific feature aggregation was achieved through the global average pooling of tumor region features per sequence, generating a 1000-dimensional latent representation. These imaging embeddings were subsequently combined with radiomic features and clinic-laboratory variables through a hierarchical feature fusion network, forming a combined, high-dimensional vector of 5718 features. This combined vector was then fed into the hierarchical feature fusion network. The fusion network is composed of multiple dense (fully-connected) layers forming a bottleneck structure (5718 features → 2048 → 512 → 512 → 64), and employs strong regularization (e.g., L2 weight decay). Furthermore, to address the dimensional disparity between modalities while preserving critical clinical information, we implemented a repeated attention mechanism that dynamically reweights clinical features at multiple network stages. This architecture compels the model to learn a compact, low-dimensional representation by automatically learning to assign low weights to redundant or uninformative features during training. This in-model, non-linear dimensionality reduction is an intrinsic property of the DL architecture and is designed to mitigate the risk of overfitting from the high-dimensional inputs. The final classification layer employs sigmoid activation for binary outcome prediction. For each biomarker prediction task, we implemented a stratified random sampling strategy: 10 PD-L1 positive and 10 PD-L1 negative patients were reserved as an independent test cohort for PD-L1 modeling, with the remaining cases undergoing four-fold cross-validation on the training cohort. To further improve the robustness and generalizability of the DL model, oversampling-based data augmentation techniques were applied to the multi-sequence MRI images. An identical approach was applied to VEGF prediction. The overall framework was illustrated in **Figure 3**.

To evaluate the contributions of automatically extracted DL features and manually designed radiomic features, we conducted two ablation experiments. The first experiment combined clinical features with raw imaging data, while the second integrated clinical and radiomic features without imaging data. All ablation models maintained identical architectural hyperparameters and training protocols to isolate modality-specific effects. Performance metrics were compared using DeLong’s test for receiver operating characteristic (ROC) curves.

### 2.7 Prognostic stratification

The entire cohort was used for prognostic analysis to maximize the statistical capability and ensure the clinical generalizability. All patients were followed up by telephone and medical record system queries. Survival outcomes were rigorously defined in accordance with RECIST 1.1 guidelines for oncology endpoints. OS was calculated from the date of pathological diagnosis to death from any causes, with surviving patients censored at last confirmed follow-up. To assess the combined predictive role of PD-L1 and VEGF, we utilized prediction scores from our multimodal framework in a Cox proportional hazards regression analysis. These scores, along with survival status and OS time, formed the basis of a Cox model predicting 1-, 2-, and 3-year OS, presented through nomograms for personalized survival predictions.

Using X-tile software (version 3.6.1, Yale University), optimal cutoff values were determined from nomogram scores to classify patients into high-risk and low-risk groups. Kaplan-Meier (KM) curves were compared with survival outcomes between these groups, with log-rank tests confirming statistical significance. To validate our stratification approach biologically, we developed a parallel Cox model using IHC-confirmed PD-L1 and VEGF status. Patients were similarly stratified, and KM curves with log-rank tests were used to compare survival outcomes. This allowed comparison between DL-based and traditional IHC-based risk stratification.

### 2.8 Statistical analysis

Statistical analyses of all patient clinical information, laboratory data, and pathological results were performed using SPSS software (version 27.0, IBM). Continuous variables were described as mean ± standard deviation (x̅ ± sd) if normally distributed; otherwise, they were reported as median (interquartile range, IQR). Independent t-tests were applied for normally distributed variables, while the Mann-Whitney U test was used for non-normally distributed variables. Categorical variables were described as numbers and percentages, and the Pearson's Chi-squared test was used for analysis. All statistical tests were two-sided, with a p-value < 0.05 considered statistically significant. ROC curves were used to evaluate the predictive performance of the model, including the area under the curve (AUC) and accuracy. Additionally, calibration curves and decision curve analysis were plotted to assess the model goodness-of-fit and clinical net benefit.

## 3 Results

### 3.1 Patient characteristics

The study cohort consisted of 96 eCCA patients, including 47 PD-L1 positive and 49 PD-L1 negative cases. The median age was 59 years (range 50–65) for PD-L1 positive patients and 58 years (range 53–62) for PD-L1 negative patients. There was no significant gender difference between the groups (p = 0.863). Tumor location was significantly different between the PD-L1 groups, with more patients in the PD-L1 positive group having distal CCA (76.6% vs. 57.1%, p = 0.043).

For VEGF expression, 46 patients were classified as VEGF positive, and 50 were classified as VEGF negative. The median age of VEGF positive patients was 56 years (range 50–62), while VEGF negative patients had a median age of 62 years (range 54–67), with a significant difference (p = 0.026). Tumor location also varied between VEGF groups, with a higher proportion of VEGF positive patients presenting with distal CCA (65.2% vs. 50%, p = 0.014). No significant differences were observed in all tumor markers and liver function indicators. Patients with PD-L1 positivity and VEGF negativity tended to have longer median survival times, though survival status and OS times did not significantly differ between the PD-L1 and VEGF groups. Baseline characteristics were summarized in **Table 2**.

**Table 2:** Baseline characteristics comparison.

Characteristic	PD-L1		*p*-value	VEGF		*p*-value
	Positive (N = 47)	Negative (N = 49)		Positive (N = 46)	Negative (N = 50)	
Age	59 (50, 65)	58 (53, 62)	0.869	56 (50, 62)	62 (54, 67)	**0.026**
Gender			0.863			0.629
Man	27 (57.4%)	29 (59.2%)		28 (60.9%)	28 (56.0%)	
Woman	20 (42.6%)	20 (40.8%)		18 (39.1%)	22 (44.0%)	
Location			0.043			0.014
Hilar CCA	11 (23.4%)	21 (42.9%)		21 (45.7%)	11 (22.0%)	
Distal CCA	36 (76.6%)	28 (57.1%)		25 (54.3%)	39 (78.0%)	
AFP	3.16 (2.27, 4.09)	3.60 (2.66, 4.55)	0.149	3.19 (2.33, 4.27)	3.43 (2.72, 4.33)	0.538
CEA	4 (3, 7)	5 (3, 8)	0.195	4 (3, 7)	4 (3, 7)	0.543
CA19-9	112 (63, 238)	152 (82, 263)	0.202	148 (68, 368)	120 (78, 233)	0.363
CA50	77 (31, 167)	119 (35, 180)	0.424	85 (33, 188)	91 (33, 156)	0.761
FER	393 (201, 669)	429 (292, 577)	0.585	440 (238, 648)	395 (281, 524)	0.809
ALT	85 (61, 131)	97 (61, 163)	0.484	92 (63, 138)	89 (60, 135)	0.671
AST	71 (54, 113)	74 (49, 95)	0.324	82 (50, 103)	67 (53, 99)	0.660
TBIL	132 (82, 210)	162 (68, 242)	0.514	165 (72, 242)	132 (75, 219)	0.405
DBIL	111 (62, 169)	127 (57, 187)	0.634	134 (54, 201)	112 (61, 168)	0.435
γ-GTP	440 (230, 760)	420 (208, 649)	0.399	465 (237, 716)	390 (164, 690)	0.397
ALP	417 (326, 555)	421 (290, 619)	0.574	476 (314, 561)	376 (269, 604)	0.104
NLR	3.69 (2.80, 4.62)	4.08 (3.00, 5.50)	0.218	4.03 (2.98, 5.50)	3.58 (2.89, 4.68)	0.450
Survival status			0.990			0.069
Death	22 (46.8%)	23 (46.9%)		26 (56.5%)	19 (38.0%)	
Censoring	25 (53.2%)	26 (53.1%)		20 (43.5%)	31 (62.0%)	
OS time (month)	26 (7, 46)	18 (7, 57)	0.939	17 (4, 49)	26 (11, 46)	0.456

Continuous variables are expressed as median (interquartile range, IQR); categorical variables are expressed as number (%).

Boldface values indicate statistically significant p-values (p < 0.05).

### 3.2 Multimodal DL framework performance

**Figure 4:**
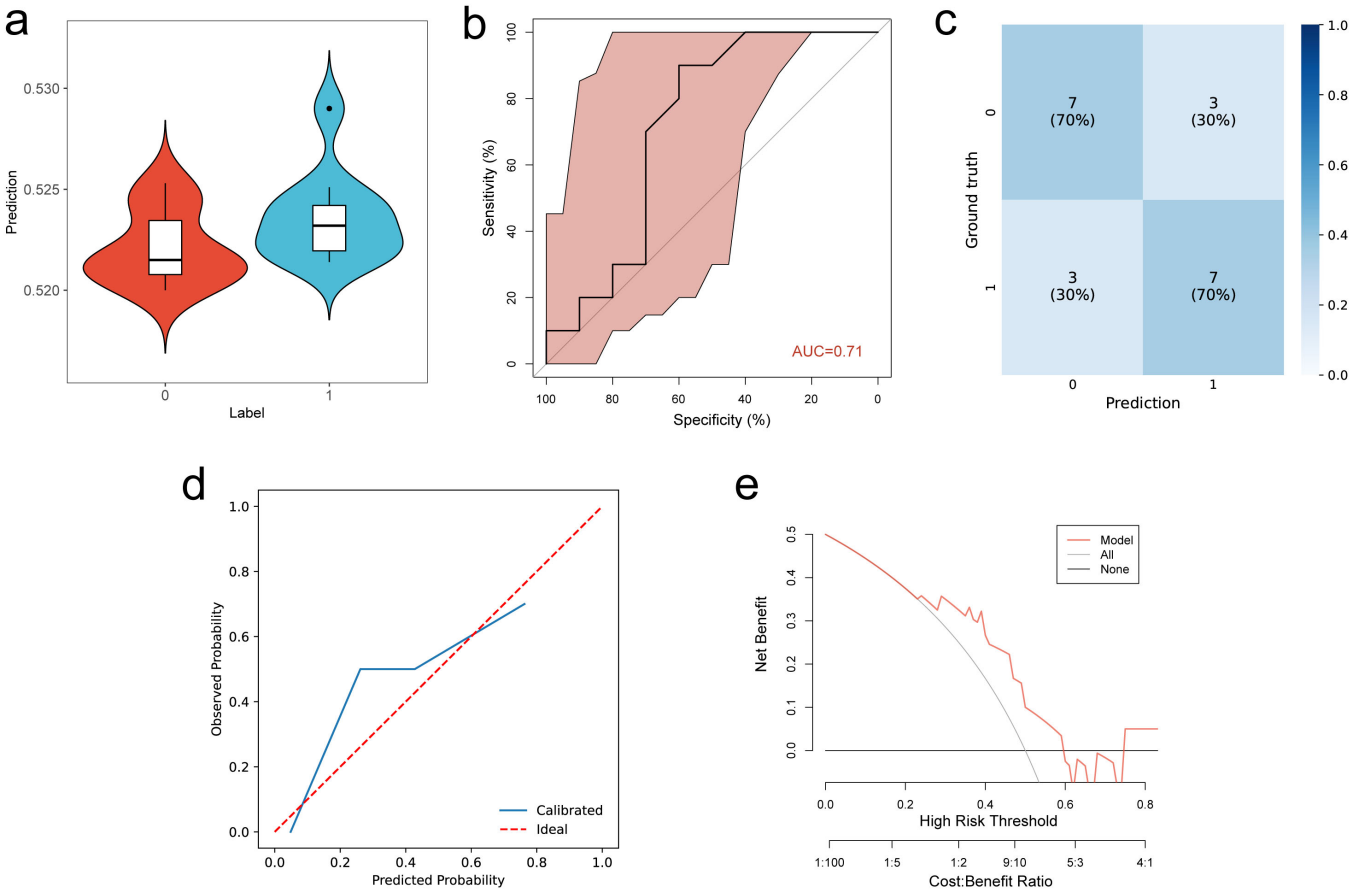
Prediction of PD-L1 status. **(a)** Violin plot showing the distribution of predicted scores for PD-L1 positive (1) and negative (0) cases. **(b–e)** Performance evaluation of PD-L1 prediction in the test cohort: **(b)** ROC curve, **(c)** Confusion matrix, **(d)** Calibration curve, **(e)** Decision curve analysis.

**Figure 5:**
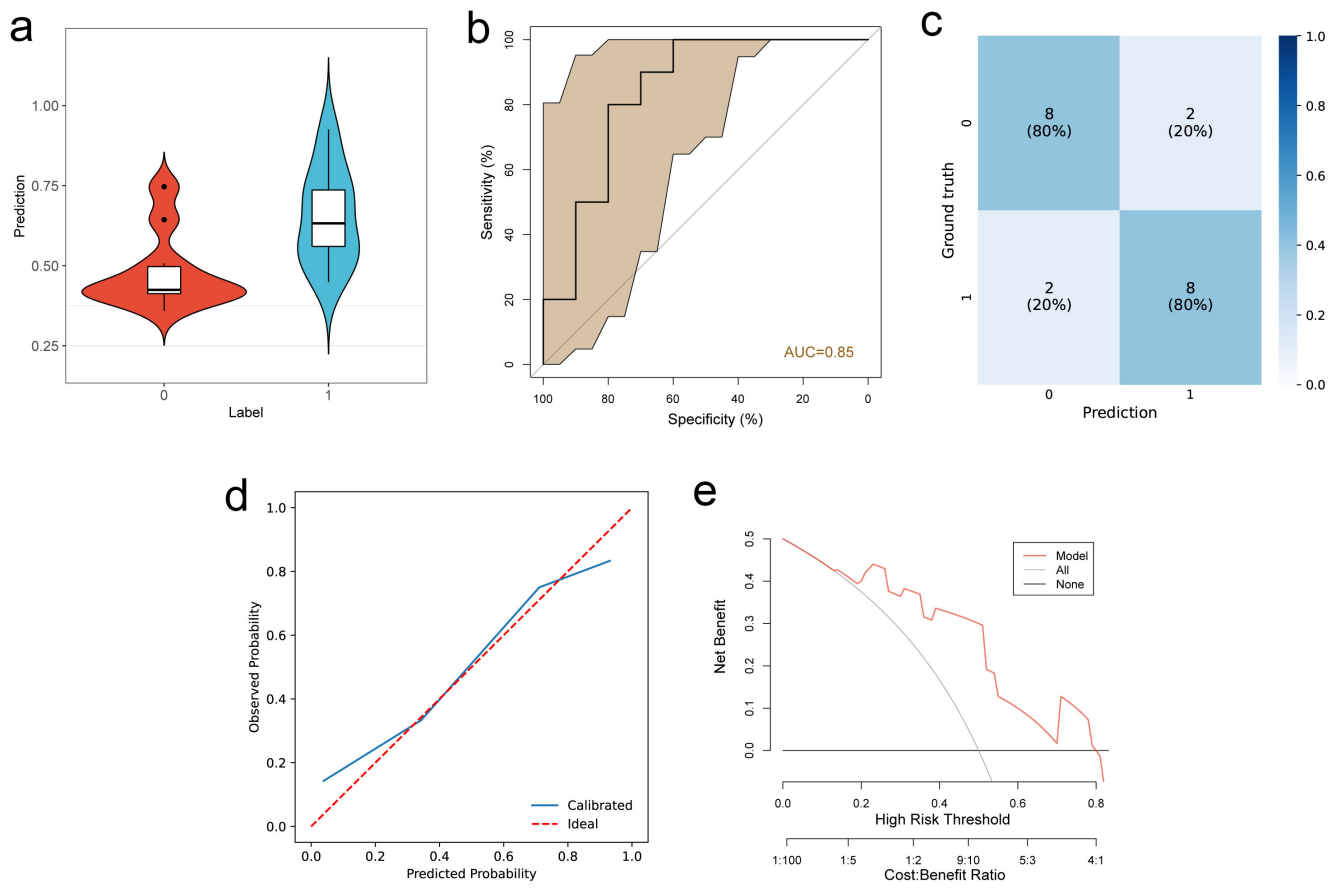
Prediction of VEGF status. **(a)** Violin plot illustrating the distribution of predicted scores for VEGF positive (1) and negative (0) cases. **(b–e)** Performance evaluation of VEGF prediction in the test cohort: **(b)** ROC curve, **(c)** Confusion matrix, **(d)** Calibration curve, **(e)** Decision curve. analysis. Calibration curve, **(e)** Decision curve analysis.

The multimodal DL model showed varying performance in predicting PD-L1 (**Figure 4**) and VEGF (**Figure 5**) expression. The ROC curve analysis revealed an AUC of 0.71 (95% CI, 0.670–0.749) for PD-L1 in the test cohort, indicating moderate performance. In contrast, the prediction for VEGF expression demonstrated higher accuracy, with an AUC of 0.85 (95% CI, 0.810–0.869) in the test cohort, reflecting strong predictive capability. In ablation experiments, the model that combined both imaging data and radiomic features outperformed models utilizing only one modality. Furthermore, DeLong’s test demonstrated that the differences between the models were statistically significant (**Table 3**). The calibration curves for the PD-L1 and VEGF tasks demonstrated good agreement with the actual outcomes, and decision curve analysis indicated that the use of the multimodal DL model nomogram provided substantial clinical net benefit across a wide range of decision thresholds.

**Table 3:** Performance comparison among models.

Modal	Cohort	PD-L1		DeLong’s test	VEGF		DeLong’s test
		AUC	Accuracy		AUC	Accuracy	
Multimodal	Validation	0.81	0.79		0.76	0.68	
	Test	0.71	0.70		0.85	0.8	
Clinical features + Imaging data	Test	0.65	0.60	<0.01	0.71	0.60	<0.01
Clinical features + Radiomic features	Test	0.63	0.55	<0.01	0.6	0.65	<0.01

The p-values from DeLong's test compare the AUC of the full Multimodal model (Test cohort) against the AUC of each ablation model (Test cohort) for the prediction of PD-L1 and VEGF, respectively.

### 3.3 Prognostic stratification and survival prediction

**Figure 6:**
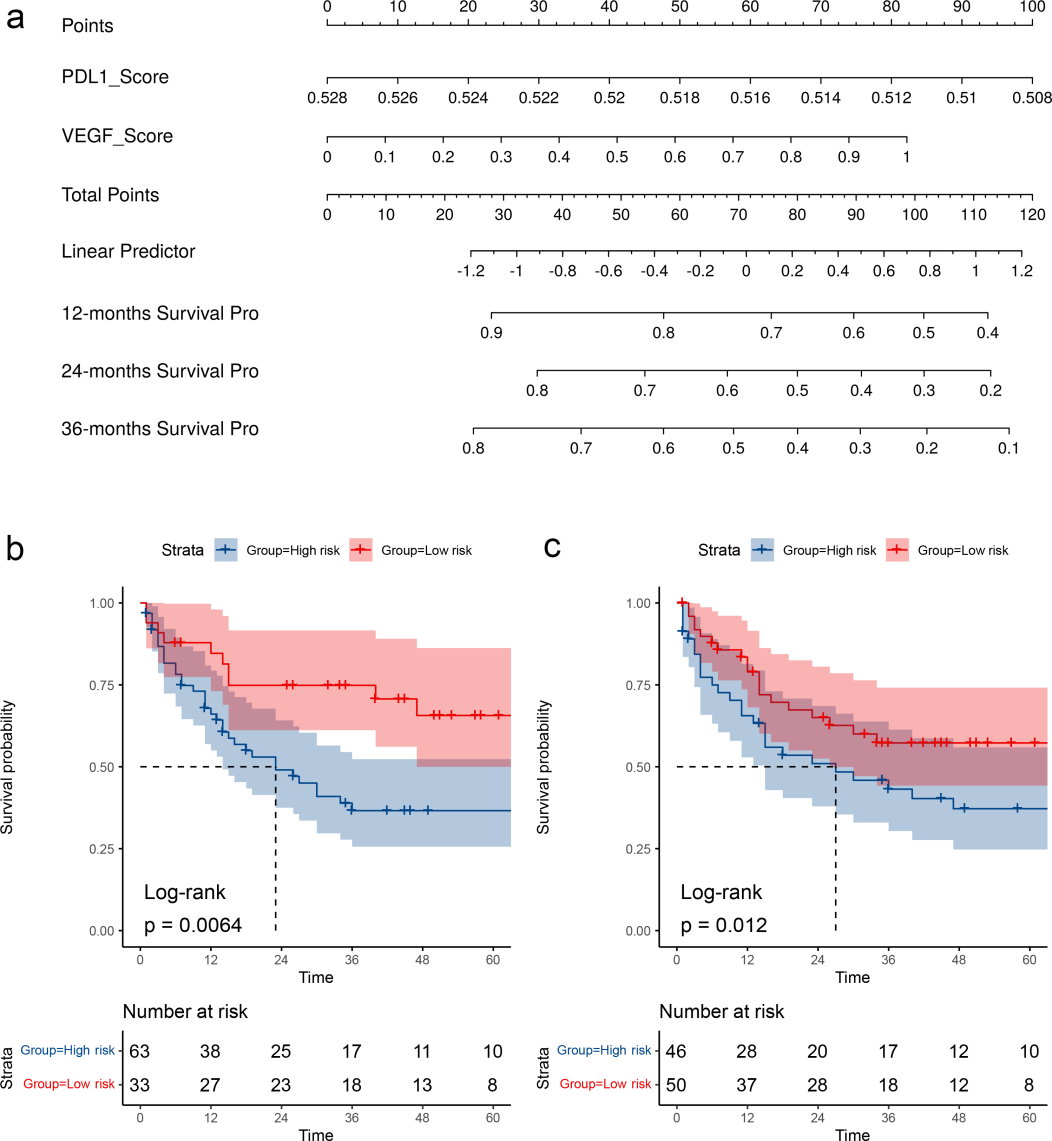
Prognostic analysis results. **(a)** Cox nomogram for individualized survival probability prediction. The “Points” axis assigns a score for a patient’s predicted PD-L1 and VEGF levels. The sum of these two scores (“Total Points”) is projected downward to obtain the “Linear Predictor” and further to the survival probability axes for 12, 24, and 36 months (Pro, probability). **(b)** Kaplan-Meier curve analysis of overall survival stratified by the risk groups derived from the multimodal deep learning framework. **(c)** Kaplan-Meier curve analysis of overall survival based on risk stratification using traditional immunohistochemistry methods.

Multivariable Cox regression analysis identified both PD-L1 (HR = 0, 95% CI: 0–0.19; p = 0.043) and VEGF (HR = 8.01, 95% CI: 1.51–42.36; p = 0.014) prediction scores as independent prognostic factors for OS. Using the constructed nomogram (**Figure 6a**) and the optimal risk stratification cutoff of 63.9, patients were categorized into high-risk (n = 63) and low-risk (n = 33) groups. KM analysis of these risk-stratified groups revealed significantly worse survival in high-risk patients (median OS: 23 months; log-rank p = 0.006) (**Figure 6b**). The IHC-based risk stratification also demonstrated comparable survival discrimination, with high-risk patients having a median OS of 27 months (log-rank p = 0.012) (**Figure 6c**).

## 4 Discussion

This study presented a multimodal DL framework that non-invasively predicts PD-L1 and VEGF expression and survival outcomes in eCCA patients by integrating DL features, radiomic features, and clinical data. Our approach achieved robust preoperative molecular profiling and reliable risk stratification. These findings highlighted the potential for integrating imaging-based analyses into personalized eCCA treatment strategies.

Emerging immunotherapies and targeted treatments have provided new therapeutic perspectives for advanced CCA. However, the benefits have primarily been observed in iCCA patients, with only a small subset of eCCA patients showing similar responses (5, 17). The main reason for this disparity lies in the significant molecular, genetic, and histopathological differences between eCCA and iCCA (12, 18). Additionally, eCCA suffers from lower rates of molecular analysis and the difficulty in identifying suitable biomarkers for rare targets (17). Our results demonstrated the feasibility of preoperative prediction for PD-L1 and VEGF status using non-invasive multimodal data, with superior performance for VEGF (AUC = 0.85) compared to PD-L1 (AUC = 0.71). The differential predictive accuracy for PD-L1 and VEGF highlights intrinsic biological and methodological challenges. VEGF's strong performance likely arises from its uniform tumor expression, which can be detected through macroscopic imaging patterns of angiogenic signaling (19). This finding aligns with several other studies on VEGF expression prediction, which consistently report AUC values exceeding 0.8 (20–23). In contrast, PD-L1’s heterogeneous expression, driven by dynamic immune-tumor interactions (24), may reduce the sensitivity of image-based models (25). Our hierarchical fusion model outperformed single-modality approaches, demonstrating the added value of combining DL-derived imaging features with handcrafted radiomics. The attention mechanism further optimized feature integration, dynamically prioritizing clinical variables like tumor location, which correlated significantly with PD-L1/VEGF positivity in our cohort. This supports recent genomic studies suggesting molecular gradients in CCA, potentially influenced by microenvironmental factors such as oxygen levels and immune infiltration (26).

Prognostic analysis identified the model-predicted PD-L1 and VEGF as significant factors for OS in eCCA patients, reflecting their important roles in tumor biology. The association between PD-L1 expression and survival may indicate a tumor microenvironment more responsive to immune checkpoint inhibition (1, 27), while VEGF-driven angiogenesis likely contributes to metastatic dissemination (9). Our DL-based Cox nomogram demonstrated risk stratification comparable to traditional IHC, effectively recapitulating biologically grounded prognostic hierarchies. The model's predictions can provide a probabilistic estimate of biomarker status when a biopsy is contraindicated, high-risk, or inconclusive. This information, while not replacing IHC, can be incorporated into multidisciplinary team discussions to inform therapeutic decisions. For example, it can assist in risk-stratifying patients or guiding the selection of first-line systemic therapies (e.g., prioritizing anti-angiogenic agents in cases with a high predicted probability of VEGF positivity). These findings suggest the nomogram’s potential to inform therapeutic decision-making, such as prioritizing high-risk patients for VEGF inhibitors like bevacizumab, while directing PD-L1-positive cases toward early PD-1/PD-L1 blockade. This approach aligns with a phase II trial evaluating the combination of anti-angiogenic antibodies and immune checkpoint inhibitors in patients with advanced CCA (28).

Despite the promising results, several limitations should be considered. First, the retrospective design and modest sample size, resulting from the low incidence and high detection cost, highlight the need for external validation in larger, prospective cohorts. Second, although the model demonstrated strong performance in VEGF prediction, further refinement is necessary for PD-L1 prediction, which remains an area of ongoing investigation. Third, the biological interpretability of DL and radiomic features remain unclear. Future studies should aim to correlate key features with spatial transcriptomics or multiplex immunohistochemistry data to enhance understanding.

## 5 Conclusions

Our study established a proof-of-concept for non-invasive, DL-driven biomarker profiling in eCCA. By transforming routine MRI into a multidimensional descriptor of tumor immune and angiogenic states, our framework could enable real-time therapeutic decision-making, particularly in resource-limited settings. As precision oncology shifts toward immune-microenvironment modulation, such tools may become indispensable for unlocking the full potential of targeted therapies in biliary tract cancers.

## Data Availability

The raw data supporting the conclusions of this article will be made available by the authors, without undue reservation.
